# The CD133^+^ Stem/Progenitor-Like Cell Subset Is Increased in Human Milk and Peripheral Blood of HIV-Positive Women

**DOI:** 10.3389/fcimb.2020.546189

**Published:** 2020-09-24

**Authors:** Jacqueline María Valverde-Villegas, Mar Naranjo-Gomez, Mélusine Durand, David Rutagwera, Anne-Sophie Bedin, Chipepo Kankasa, Ségolène Debiesse, Nicolas Nagot, Edouard Tuaillon, Philippe Van de Perre, Jean-Pierre Molès

**Affiliations:** ^1^Pathogenesis and Control of Chronic Infections (PCCI), INSERM, University of Montpellier, Établissement Français du Sang, Montpellier, France; ^2^IRMB, University of Montpellier, INSERM, CHU Montpellier, Montpellier, France; ^3^Department of Paediatrics and Child Health, University Teaching Hospital, School of Medicine University of Zambia, Lusaka, Zambia; ^4^CHU Montpellier, Department of Bacteriology-Virology and Department of Medical Information, Montpellier, France

**Keywords:** CD133, CD34, CD45, HIV-1, human milk

## Abstract

Human milk is a significant source of different CD133^+^ and/or CD34^+^ stem/progenitor-like cell subsets in healthy women but their cell distribution and percentages in this compartment of HIV-positive women have not been explored. To date, a decrease of CD34^+^ hematopoietic stem and progenitor cell frequencies in peripheral blood and bone marrow of HIV-positive patients has been reported. Herein, human milk and peripheral blood samples were collected between day 2–15 post-partum from HIV-positive and HIV-negative women, and cells were stained with stem cell markers and analyzed by flow cytometry. We report that the median percentage of CD45^+/high^CD34^−^CD133^+^ cell subset from milk and blood was significantly higher in HIV-positive than in HIV-negative women. The percentage of CD45^dim^CD34^−^CD133^+^ cell subset from blood was significantly higher in HIV-positive than HIV-negative women. Moreover, percentages of CD45^dim^CD34^+^, CD45^dim^CD34^+^CD133^−^, and CD45^+high^CD34^+^CD133^−^ cell subsets from blood were significantly lower in HIV-positive than HIV-negative women. The CD133^+^ stem/progenitor-like cell subsets are increased in early human milk and blood of HIV-positive women and are differentially distributed to CD34^+^ cell subset frequencies which are decreased in blood.

## Introduction

The percentage of activated CD4^+^ T cells productively infected by HIV-1 in the peripheral blood of asymptomatic individuals is relatively low (Simmonds et al., 1990). Thereby, the susceptibility to HIV infection and AIDS progression cannot be explained only by the consequence of a direct perturbation on mature activated immunological cells, but through non-immunologic cells including stem and progenitor cells (Re et al., 1994).

Different phenotypes of stem/progenitor cells have been described using the CD34 marker in different compartments. More recently, CD133 has taken considerable importance because it allows characterization of different stem/progenitor cell subsets when used alone or in combination with CD34, and because it describes more precisely different cell subsets which vary between organs (Handgretinger and Kuçi, 2013). For example, human cord blood (CB) or bone marrow (BM)-derived CD133^+^ cells have characteristics of primitive hematopoietic cells (Handgretinger and Kuçi, 2013), CD34^+^CD133^+^ cells of BM were enriched in primitive and myeloid progenitor cells, whereas CD34^+^CD133^−^ cells from CD45^+^ population contained B cells and late erythroid progenitors and CD34^−^CD133^+^ cells could commit to T, B, and natural killer cells lineage (Bühring et al., 1999).

In the HIV infection context, CD34^+^ hematopoietic and progenitor cells (HSPCs) were intensely investigated. Overall, CD34^+^ stem/progenitor cells are susceptible to HIV infection, an infection that impairs the differentiation and proliferation capacities of these cells (Zauli et al., 1992a,b). As a consequence, their numbers were decreased in BM and peripheral blood (PB) of HIV-positive individuals compared to HIV-negative donors (Zauli et al., 1992a,b). It was suggested that the impairment could contribute to the HIV-1/AIDS outcome by inhibiting the production of mature blood cells or BM-accessory cell populations (macrophages, fibroblasts and T cells) (Zauli et al., 1992a; Davis and Zauli, 1995; Guo et al., 2016; Bordoni et al., 2017). Subsequent studies observed that CCR5 and CXCR4, the main receptors used by HIV to infect CD4^+^ T cells, are also expressed at the surface of CD34^+^ HSPCs (Carter et al., 2011). Of note, CXCR4 was more widely expressed on multipotent CD34^+^ cells than CCR5, and its sole expression renders these cells permissive to HIV-1 infection when the sole expression of CCR5 could not (Carter et al., 2011). Furthermore, CD34^+^ cells, as well as CD133^+^ cells from BM and PB, harbored latent HIV provirus, and some authors have suggested that these cells could be potential HIV reservoirs (McNamara et al., 2013; Sebastian et al., 2017). However, there are very few studies unveiling stem/progenitor cell phenotypes and their cell frequencies assessed by CD133^+^ stem cell marker upon HIV infection. It was reported that the percentage of CD133^+^ cells from peripheral blood characterized as endothelial progenitor cells (EPCs) was increased in HIV-positive individuals with suppressed VL when compared to healthy controls (Papasavvas et al., 2012; Vecchiet et al., 2013).

In human milk (HM), stem/progenitor cells have been previously identified (Hassiotou et al., 2012; Indumathi et al., 2013). We recently identified the presence of a large and heterogeneous proportion of CD133^+^ and/or CD34^+^ stem/progenitor-like cell phenotypes from the mononuclear cell population in HM from healthy women (Valverde-Villegas et al., 2019). Also, we reported that some CD133^+^ cell subset phenotypes were far more frequent in HM than in PB. The reasons for having such a high number of stem cells in HM are unclear and it cannot be overlooked since it was hypothesized that these cells are transferred to the offspring during breastfeeding and are likely to integrate into different organs and have an active role in the development of the neonate (Molès et al., 2017, 2018). Furthermore, HIV-infected women shed both cell-associated HIV and cell-free HIV in HM which can be responsible for HIV-1 mother-to-child transmission during the breastfeeding period (Van de Perre et al., 2012; Rutagwera et al., 2019).

Taken together, our aims are to compare the distributions and percentages of CD133^+^ and/or CD34^+^ stem/progenitor-like cell subsets from HM in HIV-positive women to HIV-negative women and to its counterpart peripheral blood.

## Methods

### Design, Population, and Sample Preparation

The design of this study was a prospective observational cross-sectional study. Twenty-four HIV-infected mothers and 10 healthy mothers were recruited during pregnancy or after delivery at Mother and Newborn Hospital of the University Teaching Hospitals in Lusaka, Zambia. All participants fulfilling the inclusion criteria (aged of 18-year or more and do not suffer from serious medical events) signed an informed consent form before enrolment. Samples were collected for a consecutive period of 3 months in 2014. This study was approved by the ERES Converge institutional review board (00005948 IRB number, Lusaka) and data collection, as well as samples and, all experiments followed ethical procedures according to the Declaration of Helsinki.

Approximately, between 8–50 mL of HM from each breast and 10mL of PB samples were collected between day 2 to day 15 post-partum. Samples were centrifuged at 1,200 g for 15 min at 4°C within the next 4 h after collection to separate lactoserum and cell pellets of HM. Plasma was isolated after centrifugation of peripheral blood. Peripheral blood mononuclear cells (PBMCs) from PB were recovered from the Ficoll-plasma interface, washed three times in PBS/2% Fetal Bovine Serum. Samples were immediately cryopreserved and stored at −80°C until further laboratory analysis.

### Flow Cytometry Analyses

Cell pellets from each breast milk were thawed and pooled. Cells were stained using CD34-FITC, CD133-PE, CD45-APC Alexa-fluor700, CD38-PerCP-Cy5.5, and, a cocktail of lineage-committed antibodies conjugated with APC: CD4, CD8, CD11c, CD14, CD16, CD19, CD20 along with Live/Dead^TM^ Fixable Near-IR viability marker. Fluorescence Minus One (FMO) negative controls for CD34 and CD133 expression were included in each experiment. The cellular acquisition was performed on LSRFortessa™ cell analyzer (Becton Dickinson) and analyses were performed using FlowJo® software v10.0 (FlowJo LLC, Ashland, USA). Of note, we used the CD34-FITC, clone 581, which is recommended by the International Society of Hematotherapy and Graft Engineering (ISHAGE) for CD34^+^ cell quantification by flow cytometry (Sutherland et al., 1996), as this clone recognizes a class type III epitope and avidly binds to all glycoforms of CD34. Stem/progenitor-like cells were identified through CD34, CD133, and CD45. Details of fluorochrome-conjugated antibodies as well as the gating strategy were previously described (Valverde-Villegas et al., 2019 and see Supplementary Figure 1). Also, progenitors using lineage negative and CD38 were identified as CD34^+^CD38^−^Lin^−^ and CD34^+^CD38^+^Lin^−^ as well as CD34^+^CD133^+^Lin^−^ and CD34^+^CD133^+^Lin^−^, each one in the CD45^dim^ and CD45^+/high^ populations.

### HIV Viral Load Quantification

Viral RNAs from 560 μL of lactoserum or 200 μL of plasma were manually extracted with QIAmp® Viral RNA Mini Kit (Qiagen®, Hilden, Germany), following the manufacturer's instructions. An internal extraction control was added to the lysis buffer prior to extraction. RNAs from five HIV standards (3–7 log_10_ copies/mL), from one positive and one negative controls, were also extracted in the same batch. The qRT-PCR was performed using Generic HIV Charge Virale kit (Biocentric®, Bandol, France) following manufacturer's instructions. Amplification reaction was done using LightCycler® 480 (Roche, Indianapolis, USA) and quantification against the standard curve. The viral load in plasma and lactoserum samples were qualified as undetectable when it was below 1,000 copies/mL. Mean values of the viral load from right and left breast samples were used for analyses.

### Subclinical Mastitis (SCM): Ratio [Na^+^/K^+^] Quantification

Na^+^ and K^+^ lactoserum concentrations were measured by Compact K^+^ and Na^+^ meters according to manufacturer's recommendations (Horiba Ltd., Kyoto, Japan). Before each measurement, the calibration was done with low (150 ppm) and high (2,000 ppm) standards. The [Na^+^/K^+^] ratio >1 was indicative of SCM as suggested in a previous study (Tuaillon et al., 2017).

### Cytokines/Chemokines Measurement

Seven cytokines/chemokines, namely IL-1β, IL6, IL8, TNF-α, CXCL10, CXCL12, and IFN-γ were measured independently in lactoserum and plasma using ELISA method according to manufacturer's recommendations (PeproTech, Stockholm, Sweden). A Multiskan™ FC microplate photometer (Thermo Fisher Scientific, Vantaa, Finland) was used to measure absorbance according to manufacturer's recommendations. Mean values of cytokines/chemokines levels from the right and left breast samples were used for analyses.

### Statistical Analyses

Quantitative variables were reported as median and interquartile range (IQR). Group comparisons were performed using Mann Whitney *U*-test for non-Gaussian variables or Student's *t*-test for Gaussian variables. Percentages of CD133^+^ and CD34^+^ cell subsets of HM and its PB counterpart samples were compared between HIV-positive and HIV-negative women. Sub-analyses considering viral load were done for the HIV-positive women. In addition, correlation analyses regarding percentages of cell subsets and clinical data such as SCM, CD4^+^ T cell counts, white blood cells (WBCs), body mass index (BMI), and cytokines/chemokines measurements from mothers regarding were performed by Spearman's non-parametric correlation tests. The significance level was set at *p* < 0.05. Analyses were done using SPSS V20.0 (IBM Corp., 2011) and graphs were plotted using the GraphPad Prism 5.01 software (GraphPad Software Inc., San Diego, USA). The proportions represented in the pie chart figures was performed over the sum of median values for CD34^+^ and CD133^+^ cell subsets from mononuclear cells (CD45^−^CD34^+^, CD45^dim^CD34^+^, CD45^+/high^CD34^+^ and CD45^−^CD133^+^, CD45^dim^CD133^+^, CD45^+/high^CD133^+^). Of note, percentages of progenitor cells characterized with lineage negative (Lin^−^) and/or CD38 expression were excluded from analyses because were lower and undetectable in some samples, in both HIV-positive and HIV-negative women groups.

## Results

### Characteristics of Study Participants

Twenty-four HIV-positive participants were included in this study; 16/24 (67%) had a detectable viral load in lactoserum (right or left breast) or plasma, 11/19 (58%) received antiretroviral treatment (ART) with Atripla, and 13/24 (54%) presented subclinical mastitis (SCM). Amongst the 10 HIV-negative participants, 5/10 (50%) presented a unilateral or bilateral SCM. Demographic and clinical data of participants are shown in Table 1. There were no significant differences when HIV-negative and HIV-positive mothers were compared, except for CD4^+^ T cells counts at sampling (Table 1).

**Table 1:** Demographic and clinical characteristics HIV-positive and HIV-negative women and their infants.

	**HIV-positive (*n =* 24)**	**HIV-negative (*n =* 10)**	***p-*value^**§**^**
	**Median(IQR)**	**Median(IQR)**	**HIV+ vs. HIV–**
**Women**			
Age, years	27.0 (23.2–32.7)	23.5 (21.7–26.0)	0.07
Parity	1.0 (1.0–2.0)^*^	1.0 (1.0–2.2)	0.91
Body Mass Index (kg/m^2^)	22.6 (20.1–24.2)^*^	22.3 (21.5–24.2)	0.63
White cell counts (cells/mm^3^)^a^	7,275 (5,185–9,480)^*^	8,120 (1,374–9,870)^*^	0.84
CD4^+^ T cell counts (cells/mm^3^)^a^	479 (357–556)^*^	914 (638–1,156)^*^	**0.004**
Hemoglobin (g/dL)^a^	11.1 (10.7–12.7)^*^	10.3 (8.7–11.9)^*^	0.59
VL plasma (log)^a^^,^ ^b^	4.9 (4.0–5.7)	NA	NA
VL lactoserum (log)^a^^,^ ^c^	4.3 (3.8–5.4)	NA	NA
**Infants**			
Birth weight (g)	2,900 (1,900–3,200)^*^	2,350 (1,825–2,460)^*^	0.06

*IQR, interquartile range (25–75); VL, viral load; NA, not applicable. Bold values reached signficance*.

§ *Student's t-test (p < 0.05)*.

a *Counts at sampling*.

b *From 14 detectable women*.

c *From 13 detectable women*.

* *Missed information from 1 or 2 participants*.

### CD133^+^ Stem/Progenitor-Like Cell Percentages in HM and PB of HIV-Positive Women

**Figure 1:**
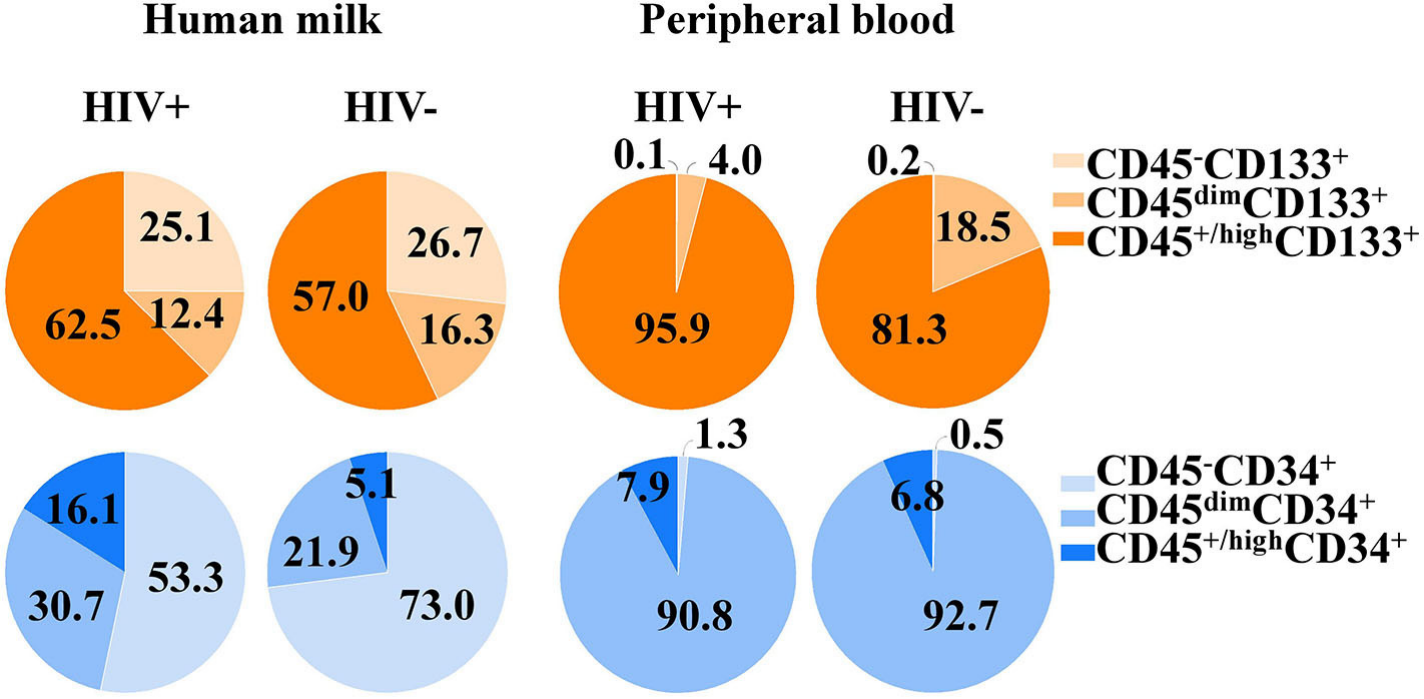
Proportions of CD34^+^ and CD133^+^ cell subsets in human milk and its peripheral blood counterpart of HIV-positive and HIV-negative women. The CD34^+^ and CD133^+^ cell subsets and cell populations according CD45 expression from both compartments.

Distribution of CD133^+^ cell subsets according CD45 expression from HM and PB compartments are represented in Figure 1. When CD133 positive expression was combined with CD34 and CD45 markers, the median percentage of CD45^+/high^CD34^−^CD133^+^ cell subset phenotype from the mononuclear cell population of HM was significantly higher in HIV-positive women than HIV-negative women (2.59% [IQR:1.02–6.13] vs. 0.92% [IQR:0.24–2.96], *p* = 0.04; Table 2). Of note, the CD45^−^CD34^−^CD133^+^ and CD45^dim^CD34^−^CD133^+^ cell subsets were also increased in HIV-positive women vs. HIV-negative women (5.1% [1.9–8.4] vs. 1.5% [1.0–8.4], *p* = 0.238 and 5.2% [2.4–9.7] vs. 2.5% [1.5–6.8], *p* = 0.179, respectively), but the difference was not statistically significant (Table 2). In PB counterpart, the CD45^+/high^CD34^−^CD133^+^ cell subset and CD45^dim^CD34^−^CD133^+^ cell subset were significantly higher in HIV-positive women vs. HIV-negative women (1.56% [0.44–5.39] vs. 0.22% [0.13–0.36], *p* = 0.004 and 0.68% [0.12–2.25] vs. 0.06% [0.02–0.38], *p* = 0.01, respectively; Table 2). Indeed, when analyzed in the CD45^−^ cell population, the median percentage of CD45^−^CD34^−^CD133^+^ cell subset was detectable in HIV-positive women while in HIV-negative women it was undetectable in some samples (0.03% [0.01–0.6] vs. 0.0% [0.0–0.1]).

**Table 2:** Percentages with interquartile range of cell subsets in HM and PB in HIV-positive and HIV-negative women.

**Cell subsets**	**HM**		**PB**	
	**HIV+ (*n =* 24)**	**HIV- (*n =* 9)**	**HIV+ (*n =* 18)**	**HIV- (*n =* 10)**
CD133^+^	1.94 (1.1–6.3)	2.96 (0.5–4.1)	1.5 (0.2–4.9)	0.24 (0.1–1.0)
CD45^−^CD133^+^	0.46 (0.1–1.1)	0.23 (0.1–2.4)	0.001 (0.0–0.02)	0.001 (0.0–0.003)
CD45^dim^CD133^+^	0.23 (0.1–0.4)	0.14 (0.1–0.4)	0.06 (0.03–0.1)	0.05 (0.02–0.1)
CD45^+/high^CD133^+^	1.16 (0.2–5.2)	0.49 (0.3–0.8)	1.37 (0.1–4.5)	0.22 (0.06–0.9)
CD34^+^	0.09 (0.03–0.3)	0.17 (0.05–0.7)	0.16 (0.1–0.3)	0.28 (0.2–0.5)
CD45^−^CD34^+^	0.04 (0.01–0.1)	0.09 (0.04–0.6)	0.002 (0.001–0.004)	0.002 (0.0–0.004)
CD45^dim^CD34^+^	0.02 (0.001–0.05)	0.03 (0.001–0.1)	**0.11 (0.1–0.2)**^**b**^	**0.26 (0.1–0.4)**^**b**^
CD45^+/high^CD34^+^	0.01 (0.0–0.03)	0.01 (0.005–0.02)	0.01 (0.004–0.02)	0.02 (0.01–0.03)
**Combined markers**				
CD45^−^CD34^+^CD133^+^	0.08 (0.0–0.7)	0.57 (0.003–0.8)	0.00 (0.0–0.01)	0.00 (0.0–0.04)
CD45^−^CD34^+^CD133^−^	0.13 (0.02–0.5)	0.64 (0.1–1.0)	0.05 (0.02–0.1)	0.15 (0.02–0.8)
CD45^−^CD34^−^CD133^+^	5.07 (1.9–8.4)	1.50 (1.0–8.4)	0.03 (0.01–0.6)	0.00 (0.0–0.1)
CD45^dim^CD34^+^CD133^+^	0.12 (0.0–0.6)	0.17 (0.0–0.7)	1.38 (1.0–3.2)	1.07 (0.3–5.7)
CD45^dim^CD34^+^CD133^−^	0.09 (0.0–0.3)	0.14 (0.0–0.9)	**2.61 (1.4–4.6)**^**c**^	**11.0 (7.4–17.5)**^**c**^
CD45^dim^CD34^−^CD133^+^	5.18 (2.4–9.7)	2.50 (1.5–6.8)	**0.68 (0.1–2.2)**^**d**^	**0.06 (0.02–0.4)**^**d**^
CD45^+/high^CD34^+^CD133^+^	0.0 (0.0–0.0)	0.004 (0.0–0.04)	0.001 (0.0–0.01)	0.004 (0.001–0.02)
CD45^+/high^CD34^+^CD133^−^	0.01 (0.0–0.03)	0.02 (0.0–0.02)	**0.004 (0.002–0.02)**^**e**^	**0.03 (0.01–0.05)**^**e**^
CD45^+/high^CD34^−^CD133^+^	**2.59 (1.0–6.1)**^**a**^	**0.92 (0.2–2.9)**^**a**^	**1.56 (0.4–5.4)**^**f**^	**0.22 (0.1–0.4)**^**f**^

*HM, human milk; PB, peripheral blood*.

a, b, c, d, e, f *Comparisons were statistically significant different (in bold) using Mann-Whitney U non-parametric unpaired tests (p < 0.05)*.

### CD34^+^ Stem/Progenitor-Like Cell Percentages in HM and PB of HIV-Positive Women

Distribution of CD34^+^ cell subsets according CD45 expression from HM and PB compartments are represented in Figure 1. When CD34 positive expression was combined with CD45 differential expression in PB, the CD45^dim^CD34^+^ cell subset was predominant and the median percentage was significantly lower in HIV-positive women than HIV-negative women (0.11% [0.1–0.2] vs. 0.26% [0.1–0.4], *p* = 0.018). When the stem/progenitor cell markers were analyzed together, the CD45^dim^CD34^+^CD133^−^ and CD45^+/high^CD34^+^CD133^−^ cell subset phenotypes were significantly lower in PB of HIV-positive women than HIV-negative women (2.61% [1.4–4.6] vs. 11.0% [7.4–17.5], *p* < 0.001 and, 0.004% [0.002–0.02] vs. 0.03% [0.01–0.05], *p* = 0.01; Table 2). On the other hand, the median percentages of these CD34^+^ cell subsets of HM were similar between HIV-positive and HIV-negative women (Table 2).

### The CD133^+^ and CD34^−^CD133^+^ Cell Subsets Are Increased in HM of Undetectable VL HIV-Positive Women

To investigate if the percentages of cell subsets could be associated with viremia, we stratified the HIV-positive individuals as undetectable VL or detectable VL (plasma or lactoserum, threshold at 1,000 copies/mL). In HM, CD45^+/high^CD133^+^, CD45^dim^CD34^−^CD133^+^, and CD45^+/high^CD34^−^CD133^+^ cell subsets were increased in undetectable VL group when compared to detectable VL group, but no significant differences were observed (Supplementary Table 1). Also, these cell subsets from PB were increased in undetectable VL group when compared to detectable VL group (Supplementary Table 1).

When these viremic or non-viremic groups were compared to HIV-negative individuals in HM, the CD45^+/high^CD133^+^ and CD45^+/high^CD34^−^CD133^+^ cell subsets were significantly higher in the undetectable VL group when compared to HIV-negative individuals (3.62% [0.64–8.84] vs. 0.49% [0.3–0.8], *p* = 0.027, and 3.97% [1.65–12.3] vs. 0.92% [0.24–2.96], *p* = 0.015; Supplementary Table 1 and Supplementary Figures 2A,B). Of note, the CD45^dim^CD34^−^CD133^+^ cell subset tended to be higher in undetectable VL group than HIV-negative individuals, but the difference was not statistically significant (7.48% [2.9–22.1] vs. 2.5% [1.5–6.8], *p* = 0.059).

### The CD133^+^ and CD34^−^CD133^+^ Cell Subsets Are Increased in PB of HIV-Positive Women With Detectable VL

In PB, the CD45^dim^CD34^−^CD133^+^ and CD45^+/high^CD34^−^CD133^+^ cell subsets were significantly increased in detectable VL group than HIV-negative individuals (0.52% [0.14–2.7] vs. 0.06% [0.02–0.38], *p* = 0.02 and 1.0% [0.45–4.0] vs. 0.22% [0.13–0.36], *p* = 0.006). Of note, these two cell subsets were also increased among the undetectable VL group when compared to HIV-negative individuals, but no significant difference was observed (CD45^dim^CD34^−^CD133^+^: 1.0% [0.08–4.6] vs. 0.06% [0.02–0.38], *p* = 0.056, and CD45^+/high^CD34^−^CD133^+^: 3.6% [0.34–7.74] vs. 0.22% [0.13–0.36], *p* = 0.056]; Supplementary Table 1). In the same way, the percentage of CD45^+/high^CD133^+^ cell subset was increased in the undetectable VL group when compared to HIV-negative group, without significant difference (3.1% [0.23–5.71] vs. 0.2% [0.06–0.9], *p* = 0.12; Supplementary Table 1).

### The CD34^+^ and CD34^+^CD133^−^ Cell Subsets Are Decreased in PB of HIV-Positive Women Independently of the HIV Viremia

In HM, no significant differences were observed when percentages of CD34^+^ cell subsets were compared between detectable and undetectable VL positive groups. The percentage of CD45^dim^CD34^+^CD133^−^ cell subset was lower in both HIV-positive groups than their counterpart PB HIV-positive groups (Supplementary Table 1). When comparisons were done against HIV-negative individuals, the CD45^dim^CD34^+^ cell subset from PB of detectable VL group was significantly decreased (0.11% [0.07–0.2] vs. 0.26% [0.14–0.45], *p* = 0.017). Furthermore, the CD45^dim^CD34^+^CD133^−^ cell subset were significantly decreased in both detectable VL group when compared to HIV-negative individuals (2.61% [1.7–4.3] vs. 11.0% [7.4–17.5], *p* < 0.001) and in undetectable VL group compared to HIV-negative individuals (2.34% [1.2–6.8] vs. 11.0% [7.4–17.5], *p* = 0.008). Finally, the CD45^+/high^CD34^+^CD133^−^ cell subset was significantly decreased in detectable VL group when compared to HIV-negative individuals (0.004% [0.002–0.01] vs. 0.03% [0.01–0.05], *p* = 0.012). All data is shown in Supplementary Table 1.

### SCM Does Not Affect the Percentages of Stem/Progenitor-Like Cell Subsets

To investigate if cell subset percentages in HM could be associated with SCM, we stratified women with or without SCM. HIV-negative women with SCM and HIV-negative women without SCM women showed no significant differences when median percentages of different cell subsets were compared (data not shown). However, the median percentages of CD45^+/high^CD133^+^ and CD45^+/high^CD34^−^CD133^+^ cell subsets were significantly higher in HIV-positive women with SCM than in HIV-negative women with SCM (Table 3). These differences were not observed when HIV-positive women without SCM were compared to HIV-negative women without SCM (Table 3). The same analysis was done for HIV VL groups and no difference was observed between the groups (Table 3). Of note, other variables such as CD4 T cell counts, white blood cells and, body mass index were analyzed but no significant association was observed with stem/progenitor-like cell subsets.

**Table 3:** Median percentages with interquartile of stem/progenitor cell subsets in HIV-positive women and HIV-negative women with SCM and non-SCM and between detectable VL and undetectable VL HIV-positive women.

**Compartment**	**Cell subset**	**SCM**		***p*-value**	**Non-SCM**		***p*-value**
		**HIV+**	**HIV–**		**HIV+**	**HIV–**	
		(*n =* 13)	(*n =* 4)		(*n =* 11)	(*n =* 5)	
HM	CD45^+/high^CD133^+^	1.6 (0.8–5.1)	0.4 (0.2–0.6)	**0.044**	0.6 (0.1–5.4)	0.5 (0.4–2.4)	1.000
	CD45^+/high^CD34^−^CD133^+^	2.7 (1.5–7.3)	0.5 (0.1–1.9)	**0.032**	2.1 (0.8–5.8)	0.9 (0.5–4.1)	0.510
		(*n =* 10)	(*n =* 5)		(*n =* 8)	(*n =* 5)	
PB	CD45^dim^CD34^+^	0.09 (0.1–0.1)	0.2 (0.1–0.6)	**0.019**	0.2 (0.07–0.3)	0.3 (0.1–0.5)	0.284
	CD45^dim^CD34^+^CD133^−^	2.3 (1.3–4.0)	10.3 (6.8–13.1)	**0.003**	3.2 (1.6–5.0)	17.2 (8.2–22.4)	**0.006**
		**SCM HIV+**			**Non-SCM HIV+**		
		**Und VL**	**Det VL**		**Und VL**	**Det VL**	
		(*n =* 4)	(*n =* 9)		(*n =* 4)	(*n =* 7)	
HM	CD45^+/high^CD133^+^	3.6 (0.9–7.7)	1.3 (0.4–3.5)	0.330	4.8 (0.4–11.8)	0.2 (0.04–4.1)	0.164
	CD45^+/high^CD34^−^CD133^+^	3.9 (1.7–7.7)	2.7 (1.0–7.3)	0.825	7.9 (1.5–19.4)	0.9 (0.2–5.7)	0.164
		(*n =* 3)	(*n =* 7)		(*n =* 3)	(*n =* 5)	
PB	CD45^dim^CD34^+^	0.1 (0.1–0.0)	0.1 (0.04–0.1)	0.383	0.1 (0.03–0.0)	0.2 (0.1–0.4)	0.571
	CD45^dim^CD34^+^CD133^−^	3.3 (1.4–0.0)	2.2 (1.0–3.7)	0.667	1.4 (0.8–1.4)	3.7 (2.5–4.9)	0.571

*SCM, subclinical mastitis; VL, viral load; Und, undetectable VL; Det, detectable VL; HM, human milk; PB, peripheral blood. Mann-Whitney U non-parametric test was applied for unpaired comparisons. Significant p < 0.05 are shown in bold*.

### Correlation of Cytokines and Chemokines From HM and PB and CD133^+^ Cell Subsets

To investigate if inflammation can influence the frequencies of CD133^+^ cell subsets, levels of TNF-α, CXCL10, CXCL12, IL-8, IL-6, IL-1β, and IFN-γ were quantified in lactoserum and plasma samples. CXCL10 and CXCL12 levels from HM were significantly increased in HIV-positive compared to HIV-negative women (148.5 [87.14–181.4] pg/mL vs. 87.90 [64.97–104.5] pg/mL, *p* = 0.008 and; 544.6 [420.6–987.8] pg/mL vs. 296.3 [276.4–365.1] pg/mL, *p* < 0.001, respectively; Supplementary Table 2). Furthermore, CXCL10 and IL-1β levels were increased in the HIV positive mothers with detectable VL compared to mothers with undetectable VL (162.6 [146.6–196.2] pg/mL vs. 90.89 [55.03–118.1] pg/mL, *p* = 0.003 and; 21.65 [12.57–30.63] vs. 11.24 [7.59–15.80] pg/mL, *p* = 0.02; Supplementary Table 2). In PB, the TNF-α, and CXCL10 levels were significantly increased in HIV-positive compared to HIV-negative women (43.08 [26.05–46.88] pg/mL vs. 22.28 [18.04–28.41] pg/mL, *p* = 0.04 and; 52.67 [15.70–67.05] pg/mL vs. 7.59 [4.80–8.55] pg/mL, *p* = 0.004, respectively; Supplementary Table 3). No significant correlation was observed between cytokine/chemokine levels and an increase of CD133^+^ cell subsets in PB of HIV-positive women (data not shown). However, IL-8 levels were significantly correlated with CD45^+/high^CD133^+^ and CD45^+/high^CD34^−^CD133^+^ cell subsets in HM of mothers with detectable VL (Supplementary Figures 2C,D).

## Discussion

Upon HIV-1 infection, cell distributions of immune cells but also of CD34^+^ stem cells are profoundly modified in PB and BM. In this study, we reported that that the progenitor/stem-like cell subsets characterized by the expression of CD133 was increased in HM of HIV-positive women as well as in their PB counterpart.

Our observations are in line with previous works, showing an increased level of peripheral circulating endothelial progenitor cells (EPCs), characterized as CD133^+^KDR^+^ cells, in HIV-positive individuals compared to healthy controls (Papasavvas et al., 2012; Vecchiet et al., 2013), but we extend the observation to other CD133^+^ cell phenotypes, less differentiated cells. It was previously observed that HIV provirus can be detected in CD133^+^ HSPCs from BM of subjects on successful ART (undetectable viral load) up to 8 years, suggesting this cell population was permissive to HIV infection (McNamara et al., 2013). Furthermore, CCR5 and CXCR4, the receptors for HIV infection, are expressed at the surface CD34^+^CD133^+^ HSPCs from umbilical cord blood and that CXCR4 was necessary for the infection by HIV-1 (Carter et al., 2011). Recently, Zaikos et al. (2018) observed proviral sequences in CD133^+^ and CD34^+^CD133^−^ HSPCs of BM and PB. They further demonstrated that these cells were the main sources of residual plasma virus as compared to other cells from BM and PB, suggesting that these HSPCs are putative reservoir of persistent HIV infection (Zaikos et al., 2018). Finally, the expression of chemokine receptors on these cells could prompt the infection by HIV and also the trafficking of HSPCs, which was observed in response to tissue damage or infections (Massberg et al., 2007). Thus, the high levels of CD45^+/high^CD133^+^ and CD34^−^CD133^+^ cell subsets in HM and PB of HIV-positive women reported in this study could be due by three mechanisms: (i) the CD133^+^ cell subsets are not (productively) infected by HIV-1 and are likely resistant to the virus; (ii) to an active self-renewing of these cells in response to the infection itself, or (iii) by active recruitment from reservoir territories through CCR5 and CXCR4 receptors toward these compartments. By contrast, the CD34^+^ cell subsets are decreased in PB of HIV-positive individuals suggesting that the mechanisms upon HIV-1 infection on CD34^+^ and CD133^+^ cells are different but are concurrent since both cell subsets seem to be resistant to HIV-1 infection.

Regarding progenitor cells through Lin^−^ and/or CD38, the cell subsets were excluded from the analyses because of the lower number of cells from both compartments. This observation suggests that most of the cell subsets reported in the CD45^dim^ and CD45^+/high^ population are lineage positive. Indeed, the CD38^+^Lin^−^ number cell subset were higher regard to CD38^−^Lin^−^ cell subset (data not shown). It was also suggested that inflammatory signals are also important for HSPCs biology, even in homeostatic conditions, thus the number of HSPCs or their proliferative condition can be influenced by inflammatory or microbial signaling, even in the absence of active infection (King and Goodell, 2011). Indeed, SCM is common in HIV-positive women during breastfeeding and pro-inflammatory chemokines/cytokines are associated with SCM in HIV-positive women (Tuaillon et al., 2017). Herein, we observed that the percentages of CD45^+/high^CD133^+^ and CD45^+/high^CD34^−^CD133^+^ cell subsets were significantly increased only in HIV-positive women with undetectable VL in HM and in HIV-positive women with SCM when compared to HIV-negative women with SCM. Because this increase of CD133^+^ cell subsets could be related to one or the other process, seven cytokines/chemokines were quantified. Only IL-8 levels from HM were positively correlated with an increase of CD45^+high^CD133^+^ and CD45^+high^CD34^−^CD133^+^ cell subsets in mothers with detectable HIV VL. On the other hand, cytokines/chemokines known to regulate the proliferation of HSPC such as IL-1β, IL-6, CXCL10, IFN-γ, TNF-α (King and Goodell, 2011; Dickinson-Copeland et al., 2016; Chavakis et al., 2019) or to control HSPC migration such as CXCL12 (Liesveld et al., 2001; Sahin and Buitenhuis, 2016; Ganuza and McKinney-Freeman, 2017) did not associate with this increase.

This study has some limitations. It is an exploratory cross-sectional study with small sample size. In a previous study, we reported differences in percentages of these cell subsets between lactation stages (Valverde-Villegas et al., 2019), however, due to the low number of samples we did not stratify the analyses according to this variable. Frozen breast milk samples were used in this study and thawing procedure has been reported to damage different cells, including CD34^+^ stem/progenitor cells. Finally, the full characterization of cell subsets regarding their stemness nature has not been done yet as well as their permissivity to HIV-1 infection assay.

In conclusion, in this study, we reported that the CD133^+^ cell subsets from early HM and PB, specifically the CD45^+/high^CD133^+^ cell subset and CD34^−^CD133^+^ cell subsets were increased in HIV-positive women. By contrast, the CD34^+^ cell subsets, specifically the CD45^dim^CD34^+^ and the CD45^dim/+high^CD34^+^CD133^−^ cell subsets were decreased in PB of HIV-positive women. Stem cell compartments as defined by one or the other marker show opposite behaviors in HM and PB in HIV infected individuals.

## Data Availability Statement

All datasets generated for this study are included in the article/Supplementary Material.

## Ethics Statement

The studies involving human participants were reviewed and approved by ERES Converge, institutional review board, Lusaka, Zambia. The patients/participants provided their written informed consent to participate in this study.

## Author Contributions

JV-V performed the experiments, provided the intellectual content and participated in the design of this work, analyzed and interpreted data, and wrote the manuscript. MN-G provided the intellectual content and analyzed and interpreted the data. MD performed some experiments and participated in analysis of data. DR enrolled participants, collected samples, recorded clinical data, and revised the manuscript. A-SB and SD performed some experiments. CK, NN, ET, and PV participated in the interpretation of data. J-PM provided intellectual content, supervised the study, participated in the design of this work, interpretation of data, and writing of the manuscript. All authors reviewed the manuscript.

## Conflict of Interest

The authors declare that the research was conducted in the absence of any commercial or financial relationships that could be construed as a potential conflict of interest.
