# Supplementary material for: The CD133+ Stem/Progenitor-Like Cell Subset Is Increased in Human Milk and Peripheral Blood of HIV-Positive Women
Source: Front Cell Infect Microbiol. 2020 Sep 24;10:546189. doi: 10.3389/fcimb.2020.546189 (PMC7546783; doi:10.3389/fcimb.2020.546189)
Supplement: Supplementary file 1 [file Image_1.pdf]

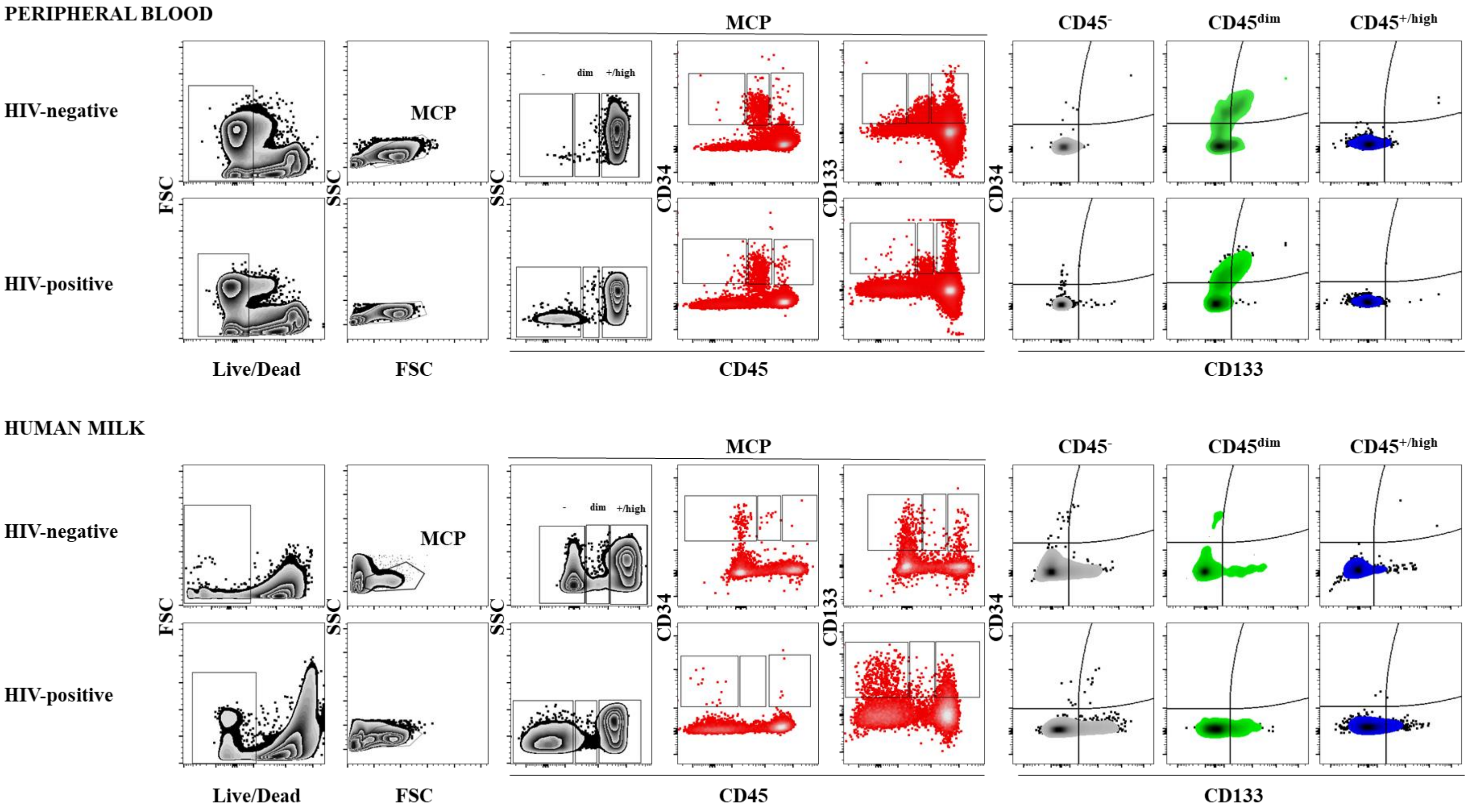

Supplementary figure 1. Gating strategy of stem/progenitor-like cells in human milk and peripheral blood. Doublets and dead cells were removed by Live/Dead viability marker positive expression and the mononuclear cell population (MCP) was identified by SSC and FSC properties. From MCP, CD45 (-/dim/+high) vs. CD34+ or CD133+ markers were gated (red); from each CD45-/dim/+high gate (zebra plot) and the combination of CD34 and CD133 were gated (grey, green and blue). Gating strategy is representative from healthy donors and HIV-positive individuals included in this study.
