# Supplementary material for: The CD133+ Stem/Progenitor-Like Cell Subset Is Increased in Human Milk and Peripheral Blood of HIV-Positive Women
Source: Front Cell Infect Microbiol. 2020 Sep 24;10:546189. doi: 10.3389/fcimb.2020.546189 (PMC7546783; doi:10.3389/fcimb.2020.546189)
Supplement: Supplementary file 2 [file Image_2.pdf]

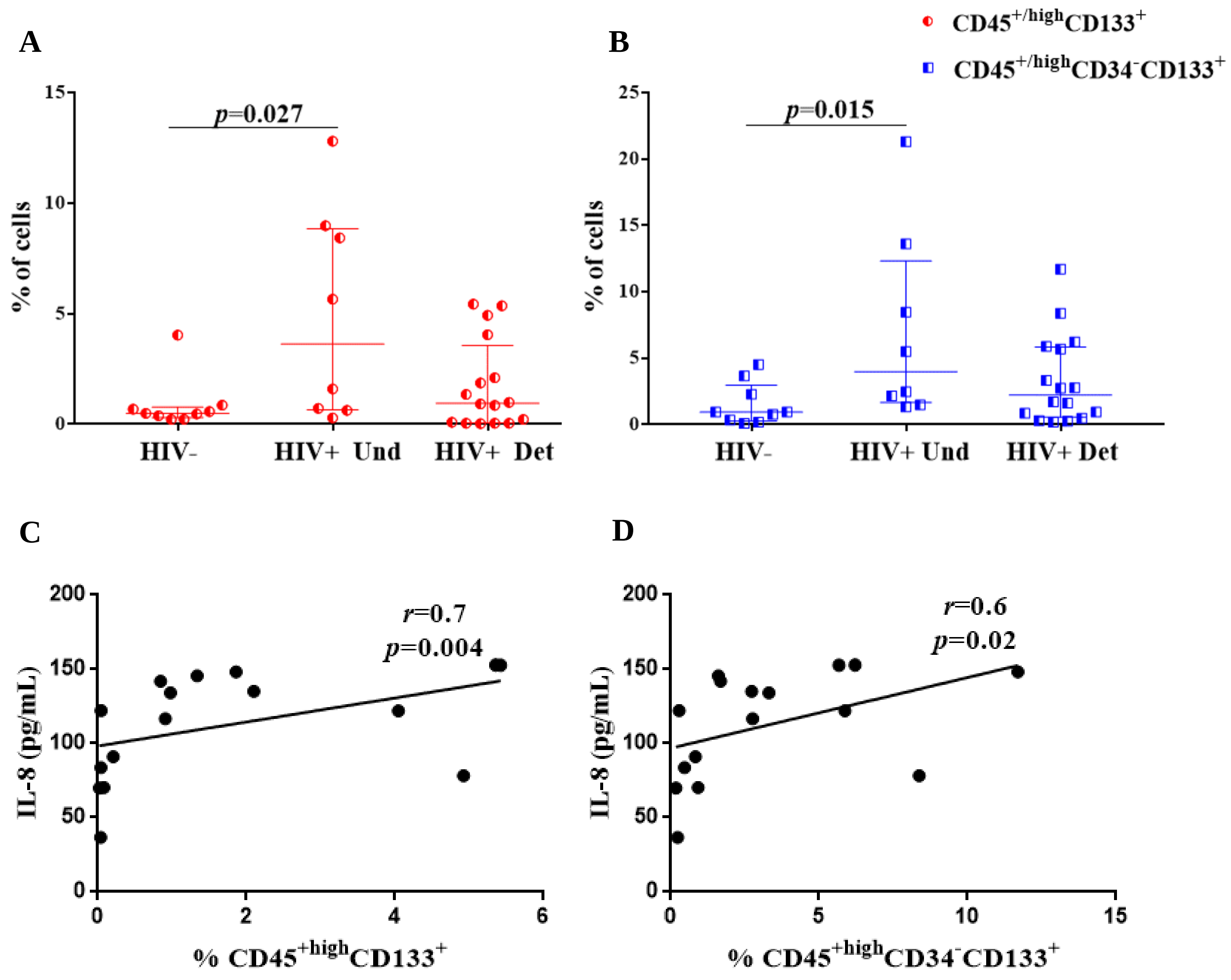

Supplementary figure 2. $CD133^{+}$ cell subsets in human milk of HIV-positive women according to HIV viremia and cytokine level. A) shows comparison of median percentages of $CD45^{+/high}CD133^{+}$ cell subset from mononuclear cells (⭘) and **B)** $CD45^{+/high}CD34^{-}CD133^{+}$ (□) cell subset from $CD45^{+/high}$ cells among HIV detectable VL women (*n*=16), HIV undetectable VL women (*n*=8) and HIV-negative women (*n*=9). Mann-Whitney U non-parametric test was applied for unpaired comparisons and significant *p*-values <0.05 are shown in bold. **C)** and **D)** show the positive and significant Spearman's correlation of $CD45^{+/high}CD133^{+}$ and $CD45^{+/high}CD34^{-}CD133^{+}$ with IL-8 levels in human milk of HIV detectable VL group (*n*=16). VL, viral load; HIV+ Und, HIV-positive woman with undetectable VL; HIV+ Det, HIV-positive woman with detectable VL.
