# Supplementary material for: The CD133+ Stem/Progenitor-Like Cell Subset Is Increased in Human Milk and Peripheral Blood of HIV-Positive Women
Source: Front Cell Infect Microbiol. 2020 Sep 24;10:546189. doi: 10.3389/fcimb.2020.546189 (PMC7546783; doi:10.3389/fcimb.2020.546189)
Supplement: Supplementary file 3 [file Table_1.pdf]

Supplementary table 1. $CD133^+$ and $CD34^+$ cell median percentages with interquartile range in undetectable and detectable VL HIV-positive and HIV-negative women.

**Compartment**	**HM**			**PB**		
**Cell subsets**	**undetectable VL**	**detectable VL**	**HIV-negative**	**undetectable VL**	**detectable VL**	**HIV-negative**
	**(*n*=8)**	**(*n*=16)**	**(*n*=9)**	**(*n*=6)**	**(*n*=12)**	**(*n*=10)**
$CD45^-CD133^+$	0.35 (0.19-1.49)	0.50 (0.13-1.13)	0.23 (0.1-2.4)	0.01 (0.00-0.03)	0.00 (0.00-0.01)	0.001 (0.0-0.003)
$CD45^{dim}CD133^+$	0.26 (0.10-1.28)	0.20 (0.02-0.45)	0.14 (0.1-0.4)	0.08 (0.02-0.51)	0.05 (0.03-0.12)	0.05 (0.02-0.1)
$CD45^{+/high}CD133^+$	3.62 (0.64-8.84)	0.95 (0.05-3.56)	0.49 (0.3-0.8)	3.11 (0.23-5.71)	0.62 (0.06-3.71)	0.22 (0.06-0.9)
$CD45^-CD34^+$	0.06 (0.03-0.12)	0.03 (0.01-0.23)	0.09 (0.04-0.6)	0.00 (0.00-0.00)	0.00 (0.00-0.01)	0.002 (0.0-0.004)
$CD45^{dim}CD34^+$	0.01 (0.00-0.08)	0.02 (0.00-0.05)	0.03 (0.001-0.1)	0.10 (0.06-0.43)	**0.11 (0.07-0.21)[a]**	**0.26 (0.1-0.4)[a]**
$CD45^{+/high}CD34^+$	0.03 (0.00-0.11)	0.01 (0.00-0.02)	0.01 (0.005-0.02)	0.01 (0.00-0.02)	0.01 (0.00-0.02)	0.02 (0.01-0.03)
$CD45^-CD34^+CD133^+$	0.35 (0.00-0.80)	0.05 (0.00-0.52)	0.57 (0.003-0.8)	0.00 (0.00-0.05)	0.00 (0.00-0.02)	0.00 (0.0-0.04)
$CD45^-CD34^+CD133^-$	0.44 (0.02-0.54)	0.10 (0.02-0.49)	0.64 (0.1-1.0)	0.06 (0.02-0.24)	0.04 (0.02-0.12)	0.15 (0.02-0.8)
$CD45^-CD34^-CD133^+$	5.69 (2.61-6.70)	4.24 (1.82-8.73)	1.50 (1.0-8.4)	0.90 (0.01-4.77)	0.02 (0.01-0.37)	0.00 (0.0-0.1)
$CD45^{dim}CD34^+CD133^+$	0.09 (0.01-3.73)	0.18 (0.00-0.39)	0.17 (0.0-0.7)	1.31 (0.77-14.48)	1.65 (0.91-2.88)	1.07 (0.3-5.7)
$CD45^{dim}CD34^+CD133^-$	0.04 (0.00-0.31)	0.12 (0.01-0.56)	0.14 (0.0-0.9)	**2.34 (1.23-6.78)[b]**	**2.61 (1.67-4.32)[c]**	**11.0 (7.4-17.5)[b,c]**
$CD45^{dim}CD34^-CD133^+$	7.48 (2.95-22.1)	4.29 (1.84-7.18)	2.50 (1.5-6.8)	1.04 (0.08-4.57)	**0.52 (0.14-2.68)[d]**	**0.06 (0.02-0.4)[d]**
$CD45^{+/high}CD34^+CD133^+$	0.00 (0.00-0.01)	0.00 (0.00-0.00)	0.004 (0.0-0.04)	0.00 (0.00-0.01)	0.00 (0.00-0.00)	0.004 (0.001-0.02)
$CD45^{+/high}CD34^+CD133^-$	0.02 (0.00-0.10)	0.00 (0.00-0.02)	0.02 (0.0-0.02)	0.00 (0.00-0.03)	**0.004 (0.002-0.01)[e]**	**0.03 (0.01-0.05)[e]**
$CD45^{+/high}CD34^-CD133^+$	3.97 (1.65-12.3)	2.21 (0.55-5.83)	0.92 (0.2-2.9)	3.64 (0.34-7.74)	**1.05 (0.45-4.00)[f]**	**0.22 (0.1-0.4)[f]**

HM, human milk; PB, peripheral blood; VL, viral load [a,b,c,d,e,f] paired comparisons were statistically significant different (in bold). Mann-Whitney non-parametric unpaired Test was used ($p<0.05$).
