# Supplementary material for: The CD133+ Stem/Progenitor-Like Cell Subset Is Increased in Human Milk and Peripheral Blood of HIV-Positive Women
Source: Front Cell Infect Microbiol. 2020 Sep 24;10:546189. doi: 10.3389/fcimb.2020.546189 (PMC7546783; doi:10.3389/fcimb.2020.546189)
Supplement: Supplementary file 4 [file Table_2.pdf]

Supplementary table 2. Cytokine and chemokine levels in human milk.

**Cytokine/ chemokine**	**HIV+** **Und VL** (*n*=8)	**HIV+** **Det VL** (*n*=16)	p-value	**All HIV+** (*n*=24)	**HIV-** (*n*=10)	p-value
TNF-α	40.7 (18.9-360.5)	57.8 (39.5-698.7)	NS	53.5 (29.4-447.3)	29.8 (18.2-285.7)	NS
CXCL10	**90.8 (55.0-118.1)**	**162.6 (146.6-196.2)**	**0.003**	**148.5 (87.14-181.4)**	**87.9 (64.97-104.5)**	**0.008**
CXCL12	507.1 (386.2-1748)	558.0 (420.6-987.8)		**544.6 (420.6-987.8)**	**296.3 (276.4-365.1)**	**<0.001**
IL-8	106.7 (57.4-115.6)	121.7 (79.10-144.4)	NS	114.2 (78.76-134.5)	96.6 (46.90-106.8)	
IL-6	34.2 (12.5-186.4)	70.4 (21.0-450.1)		50.4 (20.2-389.4)	33.8 (23.6-108.3)	NS
IL-1β	**11.2 (7.5-15.8)**	**21.6 (12.5-30.6)**	**0.02**	17.8 (11.1-24.1)	11.3 (2.7-21.7)	
IFN-γ	7.9 (5.3-9.4)	9.7 (7.8-11.0)	NS	8.9 (6.8-10.1)	5.7 (4.9-9.7)	

All values are represented in median with interquartile range (25%-75% percentile). VL, HIV viral load; Und, undetectable VL; Det[illegible] unpaired Mann-Whitney U Test was used (p<0.05) and significant values are represented in bold.
