# Supplementary material for: The CD133+ Stem/Progenitor-Like Cell Subset Is Increased in Human Milk and Peripheral Blood of HIV-Positive Women
Source: Front Cell Infect Microbiol. 2020 Sep 24;10:546189. doi: 10.3389/fcimb.2020.546189 (PMC7546783; doi:10.3389/fcimb.2020.546189)
Supplement: Supplementary file 5 [file Table_3.pdf]

Supplementary table 3. Cytokine and chemokine levels in peripheral blood.

**Cytokine/ chemokine**	**All HIV+** (*n*=7)	**HIV-** (*n*=10)	*p*-value
TNF-α	**43.0 (26.0-46.8)**	**22.2 (18.0-28.4)**	**0.04**
CXCL10	**52.6 (15.7-67.0)**	**7.5 (4.8-8.5)**	**0.004**
CXCL12	411.4 (244.9-817.0)	495.2 (316.1-796.1)	
IL-8	7.9 (3.8-8.2)	5.4 (3.9-6.7)	
IL-6	21.7 (16.6-29.8)	25.4 (17.7-30.7)	NS
IL-1β	42.6 (32.4-56.9)	36.9 (29.7-48.9)	
IFN-γ	2.5 (2.1-3.9)	2.4 (1.9-3.0)	

All values are represented in median with interquartile range (25%-75% percentile). VL, HIV viral load; Und, undetectable VL; Det, detectable VL; Mann-Whitney U non-parametric unpaired Test was used (p<0.05) and significant values are represented in bold.
